# Research Progress and Challenges in 3D Printing of Bioceramics and Bioceramic Matrix Composites

**DOI:** 10.3390/biomimetics10070428

**Published:** 2025-07-01

**Authors:** Xueni Zhao, Jizun Liu, Lingna Li

**Affiliations:** College of Mechanical and Electrical Engineering, Shaanxi University of Science and Technology, Xi’an 710021, China

**Keywords:** 3D printing, bioceramics, mechanical properties, biocompatibility, bone tissue engineering

## Abstract

Three-dimensional printing techniques can prepare complex bioceramic parts and scaffolds with high precision and accuracy, low cost, and customized geometry, which greatly broadens their application of 3D-printed bioceramics and bioceramic matrix composites in the clinical field. Nevertheless, the inadequate mechanical properties of 3D-printed bioceramic scaffolds, such as compressive strength, wear resistance, flexural strength, fracture toughness, and other properties, are a bottleneck problem and severely limit their application, which are overcome by introducing reinforcements. Three-dimensional printing techniques and the mechanical property of bioceramics and bioceramic matrix composites with different reinforcements, as well as their potential applications for bone tissue engineering, are discussed. In addition, the biological performance of 3D-printed bioceramics and scaffolds and their applications are presented. To address the challenges of insufficient mechanical strength and mismatched biological performance in bioceramic scaffolds, we summarize current solutions, including the advantages and strengthening effects of fiber, particle, whisker, and ion doping. The effectiveness of these methods is analyzed. Finally, the limitations and challenges in 3D printing of bioceramics and bioceramic matrix composites are discussed to encourage future research in this field. Our work offers a helpful guide to research and medical applications, especially application in the tissue engineering fields of bioceramics and bioceramic matrix composites.

## 1. Introduction

Three-dimensional (3D) printing, also known as additive manufacturing, is used to prepare 3D parts for diverse applications [1]. The principle of 3D printing is to utilize a computer to control materials that print parts in a layer-by-layer fashion [2], which was first proposed in 1986 [3]. Compared to the conventional technique, 3D printing can fabricate complex and customized composite parts with high precision and accurate size and geometry [4]. Bioceramics, such as hydroxyapatite (HA) [5], calcium phosphate (CaP) [6], β-tricalcium phosphate (β-TCP), α-tricalcium phosphate (α-TCP), biphasic calcium phosphate (BCP), alumina (Al_2_O_3_), zirconium dioxide (ZrO_2_) bioactive glass (BG) [7], calcium silicate (CaSiO_3_) [8], bioactive borate glass (BBG) [9], and so on, usually have unique properties, including excellent biocompatibility and osteoconductive properties [10]. They play a pivotal role in bone tissue engineering [11] and also lay the foundation for soft tissue repair and regeneration [12]. However, the limitations of bioceramics are their inadequate mechanical properties, especially fracture toughness, and so it is commonly applied for non-load-bearing conditions [13]. Among bioceramics, HA has excellent biological characteristics owing to its composition being similar to the primary inorganic component found in natural bone [14], which has been widely used as a repair and replacement material for bone grafts [15]. Unfortunately, HA cannot satisfy load-bearing applications like fracture fixation or spinal fusion due to inadequate mechanical properties [16].

Usually, bioceramic parts with complex shapes and personalized designs can hardly be fabricated through traditional techniques because the fabrication process has been limited by printing molds or manual technology [17]. A biomaterial scaffold is a 3D mesh-type component made of various types of biocompatible, bioactive, and biodegradable material, which helps to support cell adhesion and proliferation in tissue engineering [18]. Bioceramic parts produced by 3D printing can be tailored more precisely to the human body, offering numerous benefits in various applications [19]. Furthermore, 3D printing technology ensures the accurate replication of intricate details, such as the internal structures of bones or teeth. By mimicking the natural structures of the human body, bioceramics can integrate with surrounding tissues, promoting faster healing and preventing rejection reactions. The variety of bioceramic materials available for 3D printing allows for tailored compositions to meet specific tissue engineering needs. For instance, bioceramics reinforced with reinforcements can be created to provide better support for load-bearing applications like bone replacements. This customization capability ensures optimal performance and longevity of implants.

This review provides an introduction to the fabrication of bioceramics using different 3D printing techniques and highlights the enhancement of the mechanical properties of bioceramics and scaffolds. In most cases, reinforcements such as fibers, particles, whiskers, and ionic doping are incorporated in bioceramics and scaffolds, which are characterized by high mechanical properties and excellent bioactivity. These functional bioceramic composites and scaffolds have significant promise for the application of medical devices and implants, such as artificial joints, dental implants, and bone defect repair.

## 2. Three-Dimensional Printing of Bioceramics

Bioceramic parts with a similar structure to the natural bone tissue and reproducible properties can be manufactured by 3D printing techniques.

### 2.1. Direct Ink Writing (DIW)

Direct ink writing (DIW), also called robocasting, automated casting, or direct printing, uses the shear-thinning behavior of ceramic-loaded inks, which flow when being pressed but hold their shape after extrusion [20]. By moving a nozzle, it deposits these high-solid-loading inks layer by layer to build 3D scaffolds. The ink must retain its 3D shape without collapsing after being extruded. Among 3D printing techniques, DIW is capable of producing free-standing 3D components with walls of a superior aspect ratio and is characterized by low manufacturing costs, high molding efficiency, and simplicity [21,22,23]. In the process of DIW, ink flows through a syringe barrel and nozzle, is expelled from the nozzle, and then is deposited onto the underlying printed layers [24]. Compared to powder-based additive manufacturing technologies like binder-jetting, scaffolds with better mechanical properties can be printed with DIW. Additionally, the technique uses a low content of organic binders, and the composites can be easily produced. These make DIW a common and attractive technique to obtain scaffolds with macropores for biomedical applications. Lewis et al. [25] have introduced the capability of inks and provided examples of 3D ceramic structures with droplet- and filament-based DIW techniques.

The ink features can influence the printing of the ceramics by DIW. A viscoelastic ink can exhibit both viscosity and elasticity when subjected to external force, which is favorable to stable extrusion of the ink and the formation of a uniform filament in the scaffold. The ink viscosity decreases when the ink is subjected to shear forces (shear-thinning). The shear-thinning promotes the ink to be extruded smoothly through a nozzle during a DIW process, reducing the risk of nozzle blockage and filament breakage. For a bioceramic ink, a proper yield stress (the minimum stress required for the ink to start flowing) can ensure that the ink is neither too viscous to extrude nor too thin to form a molded rod in the scaffold. Moreover, the porosity and mechanical properties of bioceramic scaffolds are determined by processing parameters of DIW. Zhang et al. [26] employed DIW 3D printing to fabricate TCP bioceramic scaffolds. By adjusting the filament diameter (100–500 μm) and interlayer overlap (10–50%), they achieved a controllable porosity range of 40% to 70%. The results indicated that as porosity increased, the Young’s modulus of the scaffolds decreased exponentially. The compressive strength and modulus of the HA scaffolds through DIW were comparable to those of cancellous bone. The slurry characteristics, including rheology, particle size distribution, morphology, and wetting properties, are very important in the DIW technique. Somers et al. [27] fabricated microporous scaffolds with the doped β-TCP in ink. Compared with the undoped scaffolds, the compressive strength and density of the doped scaffolds were significantly improved via DIW. Based on optimized slurry characteristics in DIW, on-demand customized bone substitutes were developed via DIW and can be implanted in a load-bearing site.

Yang et al. [28] prepared the Al_2_O_3_ green body impeller via DIW and thermally induced solidification. When the appropriate content of carrageenan was introduced to the ceramic slurry, the viscosity of the slurry increased at 55 °C. An optimized paste with 0.4 wt% carrageenan could solidify rapidly. The intricate-shaped engine blade samples with a nearly pore-free structure would not collapse. Al_2_O_3_ ceramic parts with fine and complicated features and inclined planes can be successfully prepared with DIW. Carloni et al. [29] fabricated transparent Al_2_O_3_ ceramic parts by 3D printing. The 3D-printed samples had nearly full density and transparency through post-processing steps such as debinding, vacuum sintering, and polishing. The density of Al_2_O_3_ ceramics can be improved after two-step vacuum sintering, and the highest-quality 3D-printed transparent ceramics were obtained. Li et al. [30] fabricated ZrO_2_ biological scaffolds using DIW with a 70 wt% water-based ink. The as-printed scaffolds showed a compressive strength of 8 MPa, and the sintered scaffolds achieved 63% porosity, making them suitable for tissue engineering applications. The density and mechanical properties of printed bioceramic parts were improved by vacuum sintering, laser sintering, two-step microwave sintering, and spark plasma sintering to a certain extent [31,32,33,34]. The addition of sintering additives can significantly improve the densification of bioceramic parts and osseointegration properties. It has been reported that incorporating additives can significantly enhance the sintering process and effectively reduce the sintering temperature [35]. Al_2_O_3_ ceramics with the addition of TiO_2_ effectively reduced the sintering time and energy consumption during the process. The densification and strength of Al_2_O_3_ ceramic parts were also improved by adding TiO_2_ [36]. Dense structures with high mechanical properties and complex shapes can be obtained by the post-processing of the printed bioceramics. The biological TiO_2_ scaffolds were fabricated by direct writing, offering the advantage of smaller feature sizes, simpler operation, and greater flexibility [37]. TiO_2_ scaffolds exhibited excellent biocompatibility and bioactivity, which were conducive to cell growth and adhesion.

Although there are many advantages of DIW, such as low manufacturing costs, flexibility of manufacturing, and equipment requirements that can be easily obtained, the parts manufactured by DIW generally need to be subsequently sintered and cured. Some bioceramics cannot be printed with DIW because of the high sintering temperature and extended debinding time in the process of the post-treatment after DIW. Additionally, the cracking and collapse of bioceramic parts can occur, which is caused by the decomposition and release of organic components during processing [38]. Porosity and cracks with heat treatments also influence the densification and mechanical performance of bioceramics [39] and can be reduced by increasing solid loading in an ink. Unfortunately, it is difficult to print bioceramics with high solid loading due to a lack of proper slurry with a low viscosity and high solid content for fabricating bioceramic green bodies in the direct writing molding process. The influence of morphology and structure of ceramic powder on slurry characteristics, forming, and printing need to be systematically investigated to obtain bioceramic scaffolds with high solid loading.

### 2.2. Fused Deposition Modeling (FDM)

Fused Deposition Modeling (FDM) is one of the most widely used 3D printing technologies, where a material in filament form is melted by a heater and extruded through a nozzle. Common materials for FDM include ABS, PC, PLA, PCL, and PPSF. This technique can also be used to process bioceramics and bioceramic matrix composite scaffolds. Bioceramic scaffolds comprising bioactive glasses or calcium phosphates exhibit a notable level of biocompatibility and possess the ability to induce bone growth and facilitate bone regeneration. The compressive strength of bioceramic scaffolds using FDM and slip casting achieved 16–18 MPa, which was higher than human cancellous bone [40]. The optimum compressive strength can be achieved when porosity was controlled within a certain range, which can promote the ingrowth of bone cells [41]. Song et al. [42] produced hierarchical scaffolds featuring customized macro/microporous architectures by FDM. The scaffolds, incorporating macropores (100–800 μm) and micropores (2–10 μm), were successfully created through the technique, which laid a good foundation for application in bone tissue regeneration. Glass-infiltrated Al_2_O_3_ scaffolds exhibited good translucency, and the mechanical properties were close to pure alumina through FDM. In addition, FDM printers also offer advantages such as being an energy-efficient process, a low-cost process with minimal material waste, and an easy and economical way to prepare customized dental ceramic restorations with various types so that it can be applied for bone restoration [43].

There are some challenges to consider when printing bioceramics using FDM [44]. The selection of suitable bioceramic materials, binders, and sacrificial materials is crucial to ensuring biocompatibility and obtaining excellent mechanical properties. The temperature and speed of printing must be carefully optimized to avoid material degradation or warping. The use of FDM may introduce certain polymers, such as ABS, PC, and so on, potentially leading to reduced bioactivity in bioceramics. The printed bioceramics often exhibit problems such as interlayer interfaces, voids, and surface roughness, which have an adverse impact on their overall quality and performance.

### 2.3. Digital Light Processing (DLP)

**Figure 1:**
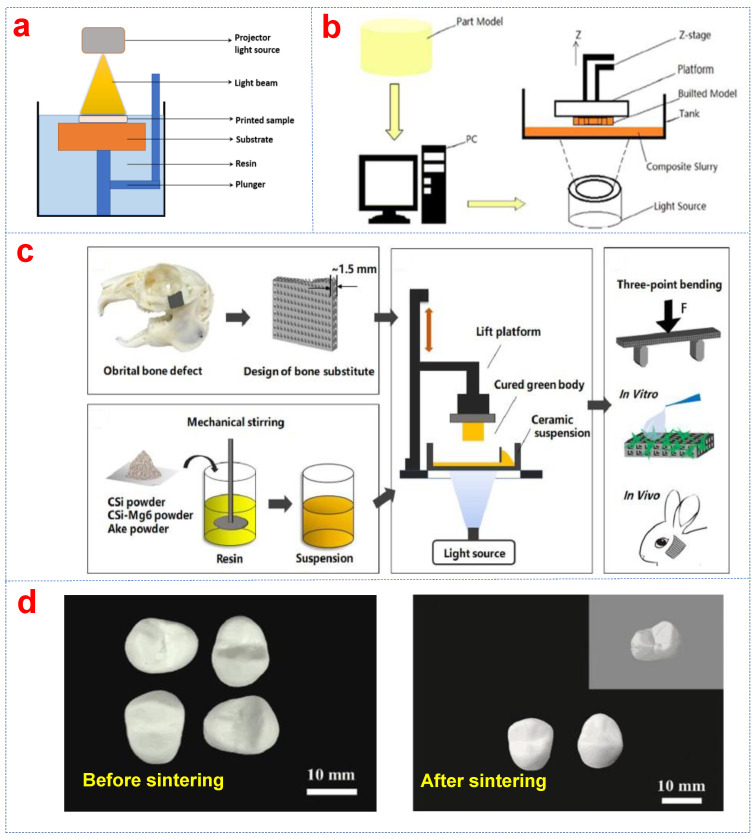
(**a**) Diagrammatic sketch [46] and (**b**) principles of DLP [47], (**c**) bioceramic scaffold via DLP for in situ orbital reconstruction [49], (**d**) ZrO_2_ teeth before and after sintering [68].

In recent years, bioceramic parts have been manufactured using stereolithography-based additive manufacturing due to its low cost, high accuracy, and short cycle time [45]. There are several technological variants (DLP, stereolithography (SLA), and others) of vat photopolymerization that can be used to print bioceramics [39]. The schematic diagram and principles of DLP are shown in Figure 1a,b [46,47].

In comparison with the conventional stereolithography point-line-layer scanning process, bioceramic components can be printed with high efficiency via DLP [48]. Furthermore, the size of the horizontal section of the bioceramic parts or the complexity of the structure had little impact on the molding speed of DLP. The ultrathin bioceramic scaffolds with high-size precision pores can also be developed. The schematic illustration is shown in Figure 1c. The 1.5 mm thick scaffolds with more than 60% porosity were constructed by DLP technology [49]. The scaffolds had significant osteogenic activity and specific shapes for bone repair.

**Figure 2:**
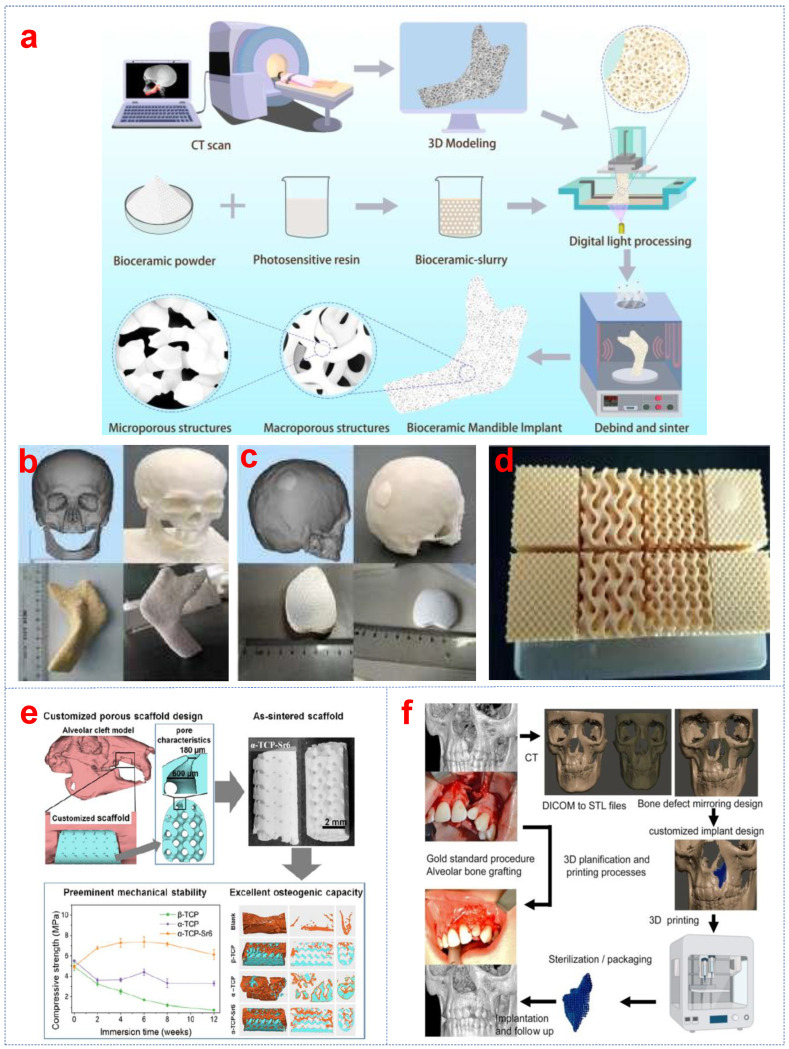
(**a**) The fabrication of the patient-specific β-TCP bioceramic bone scaffold and the customized β-TCP bioceramic bone scaffold for the repair of (**b**) a large-scale defect in the mandible and (**c**) a crania defect, (**d**) β-TCP scaffold parts with distinct pore configurations prepared by DLP [53], (**e**) the design and fabrication of alveolar cleft porous scaffold [56], and (**f**) the flow chart for a patient with a left alveolar cleft [57].

**Figure 3:**
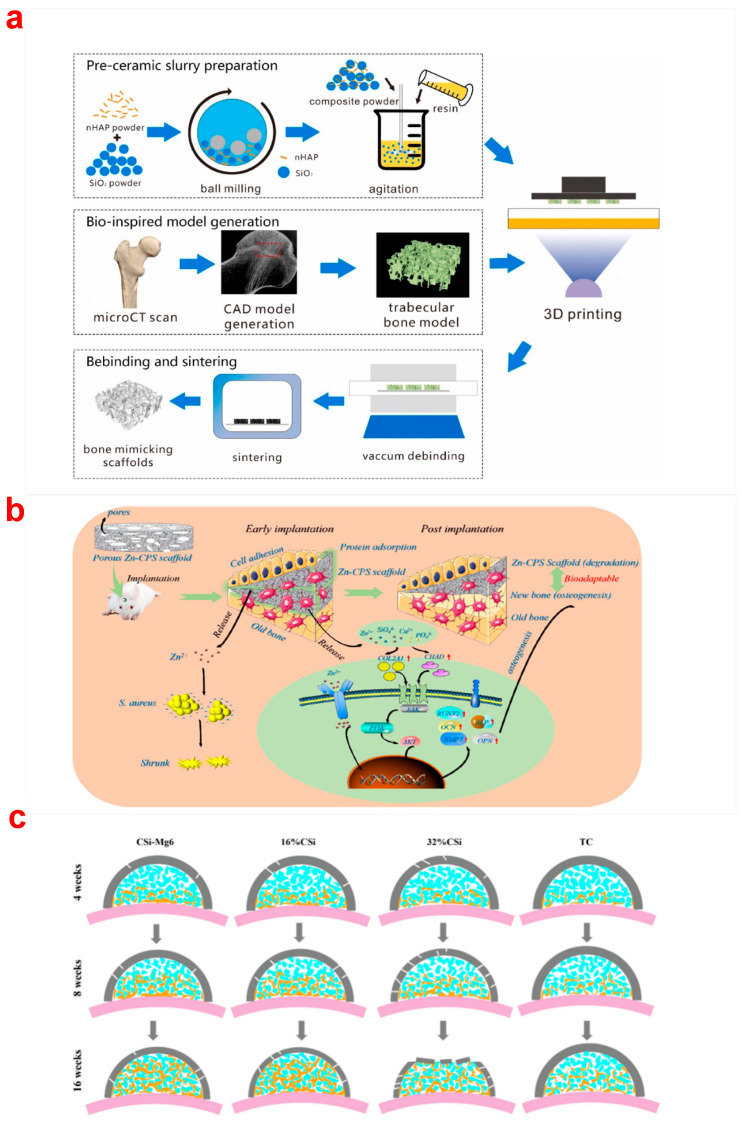
(**a**) The fabrication process of the trabecular mimicking silica-doped scaffolds by DLP [62], (**b**) the mechanism of Zn-CPS bioceramics stimulating bioadaptability [64], (**c**) a schematic representation of the biodegradation process and new bone growth in CSi/CSi-Mg6 scaffolds compared to titanium mesh. Blue, bone grafts; yellow, new bone; grey, scaffolds; pink, original bone [65].

HA, one of the most studied calcium phosphate bioceramics in bone tissue engineering, positively influences osteoblast adhesion and proliferation due to its chemical similarity to natural bone mineral [50]. This property enables the design of HA with tailored surface structures and charge to optimize material behavior in biological environments [51]. After HA scaffolds prepared through DLP are implanted, they will gradually degrade, and new tissues will form as the healing process progresses. SEM morphology illustrates that the distribution of cells increased with increasing culture days (1, 3, and 5 days) [52]. The cells formed connections with and covered the scaffold. Moreover, cells grown on the porous scaffold displayed an increased amount of calcium deposition. The surface of the sample exhibited faster cell proliferation. The precision of porous scaffold structures can be efficiently regulated with the help of DLP. Another common calcium phosphate ceramic is TCP, which is widely studied due to its rapid degradation rate and ability to form strong bone-ceramic bonds. TCP ceramics have demonstrated higher biodegradability than other biomaterial implants. The primary mechanism of TCP bioactivity involves the partial dissolution and release of calcium (Ca) and phosphate ions both in vitro and in vivo, leading to bioapatite precipitation on the bioceramic scaffold surface. β-TCP is the most advantageous form for TCP scaffolds due to its mechanical strength and chemical stability. β-TCP scaffolds with regulated pores were fabricated via DLP. The fabrication process of a patient-specific β-TCP scaffold is shown in Figure 2a [53]. The compressive strength (20–30 MPa) and elastic modulus (2–4 GPa) were increased compared with those (1.60 MPa and 513 MPa) in the previous studies [54]. The personalized β-TCP bioceramic scaffolds can be applied to treat large-scale mandible and crania defects in patients (Figure 2b,c). Green bodies with cured and dense pore struts exhibited highly interconnected pores and had few deformations or other defects via DLP printing (Figure 2d). The β-TCP bone scaffold with excellent biocompatibility was fabricated by DLP technology, which can promote the proliferation, adhesion, and differentiation of osteoblasts [55]. The viability of the bone marrow mesenchymal stem cells (BMSCs) remained above 90% from day 1 to day 3, which indicated that the scaffold exhibited minimal cytotoxicity. The confocal laser microscopy images of cells and scaffold show that the number of cells adhered and proliferated along the pore structure increased with the extended culture time. The α-TCP bioceramic scaffolds with pore sizes (300 μm–1 mm) demonstrated high fidelity and accuracy. They can be constructed and applied in bone tissue regeneration (Figure 2e,f) [56,57]. In addition, CaP bioceramic scaffolds with custom-made precisive structures were manufactured with high-resolution DLP technology and can be used for remote isolation bone regeneration [58]. Recently, Liu and colleagues used akermanite (Ca_2_MgSi_2_O_7_) as an alkaline bone implant for treating osteoporosis and achieved promising results. Their findings showed that akermanite performed better in bone regeneration during osteoporotic bone defect healing compared to β-TCP and Hardystone [59]. Liu et al. [60] compared the proliferation and osteogenic differentiation capabilities of stem cells on akermanite and β-TCP. The study indicated that human adipose-derived stem cells (hASCs) attached to and proliferated on akermanite ceramics in a similar way to β-TCP. Akermanite ceramics exhibit good in vitro osteogenic induction bioactivity, making them a potential scaffold for bone regeneration in tissue engineering applications. Hydroxyapatite-akermanite-Fe_3_O_4_ bioceramic scaffolds with controllable photothermal performance can be successfully fabricated [61]. The silica-doped HA ceramics prepared by DLP possessed a structure similar to trabecular bone (Figure 3a) [62]. The scaffolds are biodegradable and do not have potential cytotoxicity to cells.

In addition to calcium phosphate bioceramic scaffolds and calcium silicate bioceramic scaffolds, other bioceramic scaffolds by DLP also possess excellent biological properties. For example, the biological activity and mechanical properties of porous ZrO_2_ scaffolds were enhanced with the addition of calcium silicate and HA [63]. The incorporation of additives also influences the bioactivity of bioceramics. Zinc-containing silicocarnotite (Zn-CPS) scaffolds exhibited good antibacterial capacity, osteogenesis, and degradation [64]. The mechanism of Zn-CPS scaffolds stimulating antibacterial properties and bioadaptability is shown in Figure 3b. The protein absorption of CPS can be promoted by adding ZnO, and the antibacterial and osteogenesis ability of Zn-CPS can be enhanced when the content of ZnO increases. The macroporous dome-like meshes with varying mass ratios of CSi/CSi-Mg6 were prepared by DLP using wollastonite bioceramic without and with 6% magnesium addition (CSi, CSi-Mg6) [65]. The biodegradation process and the new bone growth of the CSi/CSi-Mg6 scaffolds and the titanium mesh are shown in Figure 3c. HA bone grafts degraded slowly when extending the implantation time, providing a certain spatial maintenance for further bone growth, while the degradability of bioceramic scaffolds containing 16% CSi can be significantly improved, and the scaffolds can accelerate the growth of new bone. The biodegradability of bioceramic scaffolds can be greatly improved with the addition of baghdadite [66].

As one of the most common bioceramics, ZrO_2_ ceramics possess high bending strength, toughness, chemical resistance, and biocompatibility [67]. The sintered ZrO_2_ all-ceramic teeth had a dense structure (Figure 1d) [68]. Their Vickers hardness was 12.6 GPa, and the fracture toughness was 5.25 MPa∙m^1/2^, which were similar to the one fabricated by dry pressing [69]. There are no obvious internal processing defects or cracks on the surface of the ZrO_2_ ceramics.

Post-processing after printing is necessary for bioceramic scaffolds and components after DLP printing. The density and strength of bioceramic parts increased after degreasing and sintering [45,70]. The porous akermanite bioceramics with over 80% porosity had improved compressive strength (more than 2 MPa) after being sintered at 1100 °C [71]. The compressive strength of sintered scaffolds with 60% elongation was 11.51 ± 1.75 MPa, which was higher than that of those with 0, 20%, and 40% elongation. Although the mechanical performance and density of bioceramics improve after post-processing, their mechanical properties are still poor and can only satisfy the requirement of repairing cancellous bone defects.

Bioceramic scaffolds produced through DLP methods display favorable mechanical properties and bioactivity, which include effective bone-inducing and blood vessel-forming capabilities. However, the inherent bioactivity of bioceramics can be reduced by additives such as dispersants and photopolymerizable resins, preventing the materials from achieving their optimal biological performance. These challenges emphasize the necessity of improving material compositions and manufacturing processes, through which key factors (including printability, structural integrity, and biological functionality) in DLP-based bioceramic scaffold production can be better balanced.

### 2.4. Stereolithography (SLA)

**Figure 4:**
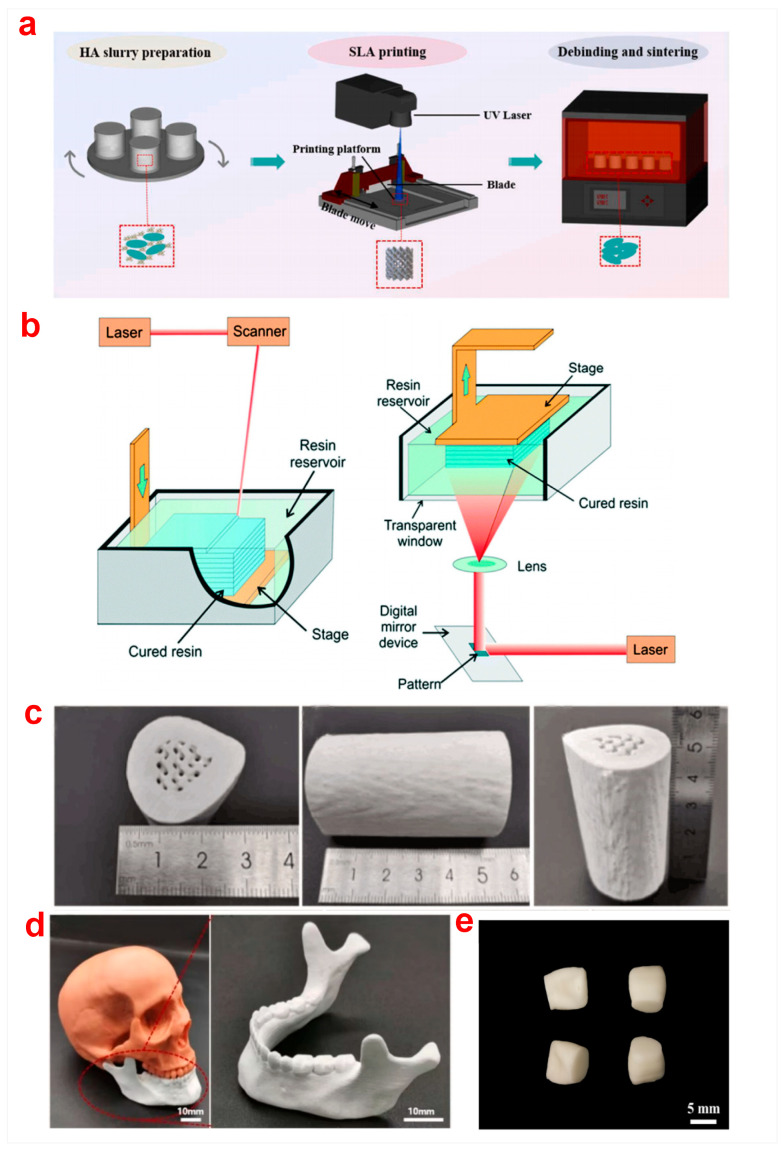
(**a**) The SLA process of an osteoid ceramic, (**b**) the stereolithography technique [72], (**c**) an osteoid HA bioceramic of large segment with different orientation views, (**d**) a customized HA mandible [74], and (**e**) Al_2_O_3_-ZrO_2_ dental implants with SLA [76].

As a kind of photocuring and the most mature technique of additive manufacturing, SLA printing of bioceramics includes the fabrication of the slurry, printing, debinding, and sintering processes. The osteoid ceramic design and the SLA 3D printing process are shown in Figure 4a,b [72]. Compared with traditional methods, SLA can print components with large size, complex structure, high precision, and fine size, which has advantages for the flexibility of printing ceramic parts and the easy control of geometry [73]. HA bioceramic scaffolds with personalized, large, and osteoid structures were printed via SLA, which laid the foundation for blood vessel growth and large bone defect repair [74]. An optimized SLA printing process was successfully utilized to fabricate HA osteoid ceramics with approximately 54 mm in length and 30 mm in width that accurately simulate the structure of the human femur (Figure 4c). SLA printed complex parts such as a precise and compact school emblem, a cage without natural bone grafting, and a customized HA mandible (Figure 4d). Similarly, porous scaffolds with complexity and good interconnectivity were prepared via SLA, which allows for human umbilical vein endothelial cells to be evenly distributed and multiplied on scaffolds [75].

Al_2_O_3_-ZrO_2_ bioceramics are non-cytotoxic and have outstanding mechanical properties and biocompatibility. The surface of Al_2_O_3_-ZrO_2_ dental implants prepared through SLA was dense and smooth and free of cracks and delamination, as shown in Figure 4e [76]. Liquid precursor infiltration, in situ precipitation, and SLA were integrated to introduce the second phase and decrease the sintering temperature to obtain Al_2_O_3_-ZrO_2_ composites with an elastic modulus of 188–318 GPa [77,78]. Most bioceramic scaffolds have strength in the range of natural skull bone. The study by Lu et al. [79] showed that the pore geometry of calcium silicate scaffolds can be designed through SLA and influenced the mechanical and structural stability of the scaffolds. CaSiO_3_ bioceramics have better osteoinductivity, biological activity, and osseointegration capabilities compared with CaP bioceramics. They can release calcium (Ca^2+^) and silicon (Si^4+^) ions in body fluids, which induce the deposition of hydroxyapatite (HA) and form chemical bonds with the host bone, thereby promoting bone repair. The bioactivity, degradability, and bone regeneration potential of calcium silicate-based ceramics highlight their potential as third-generation biomaterials [8], which can promote regenerative responses in natural human tissues, such as the ability to induce osteogenesis, similar to hydroxyapatite [80]. Similarly, β-TCP bioceramic scaffolds with gyroid structures similar to cancellous bone were produced through SLA. The scaffolds exhibited excellent mechanical strength and cell proliferation in comparison to grid-like bioceramic scaffolds [81].

Liu et al. [82] prepared the HA scaffold with different pore sizes of 400, 500, and 600 μm through SLA. The HA scaffold had an enhanced compressive strength as the pore size increased. BCP bioceramics can significantly promote the proliferation of MC3T3-E1 cells through the release of Ca and PO_3_ ions while demonstrating excellent bioactivity [83]. HA is integrated with different calcium phosphate compositions to regulate mechanical and biological properties, further improving its bone regeneration performance [84]. BCP ceramics, which combine HA and β-TCP, play a vital role in bone tissue regeneration due to their biocompatibility, bioactivity, and osteoconductivity [85]. Compared to pure HA and pure β-TCP scaffolds, BCP scaffolds exhibit controlled degradation rates, better biocompatibility, and enhanced bone regeneration capacity [86]. The compressive strength of the BCP scaffolds was similar to that of cortical bone after sintering at 1250 °C [87]. The structure and size of the scaffold can be precisely controlled with SLA, and surgical procedures such as implant placements and complex surgeries can be accelerated and improved [83]. SLA can print HA parts with high precision using aqueous HA suspension with low viscosity, high curing depth, and small curing width. The chemical composition of HA with a complex structure was similar to that of the original HA powder [57].

Post-processing, such as the cleaning, curing, and finishing of printed parts, can heavily influence the performance of bioceramics produced with SLA. The residual tensile stress of ZrO_2_ ceramics was effectively regulated by the sintering temperature, holding time, and heat rate of the degreasing–sintering process [88]. The mechanical properties and density of bioceramics can also improve by changing sintering parameters. Densification and the density of bioceramics are affected by the degree of particle packing, solid content of the initial ink, and sintering atmosphere in post processing [89]. In addition, the phase of scaffolds can also influence mechanical properties of bioceramics.

### 2.5. Selective Laser Sintering (SLS)

**Figure 5:**
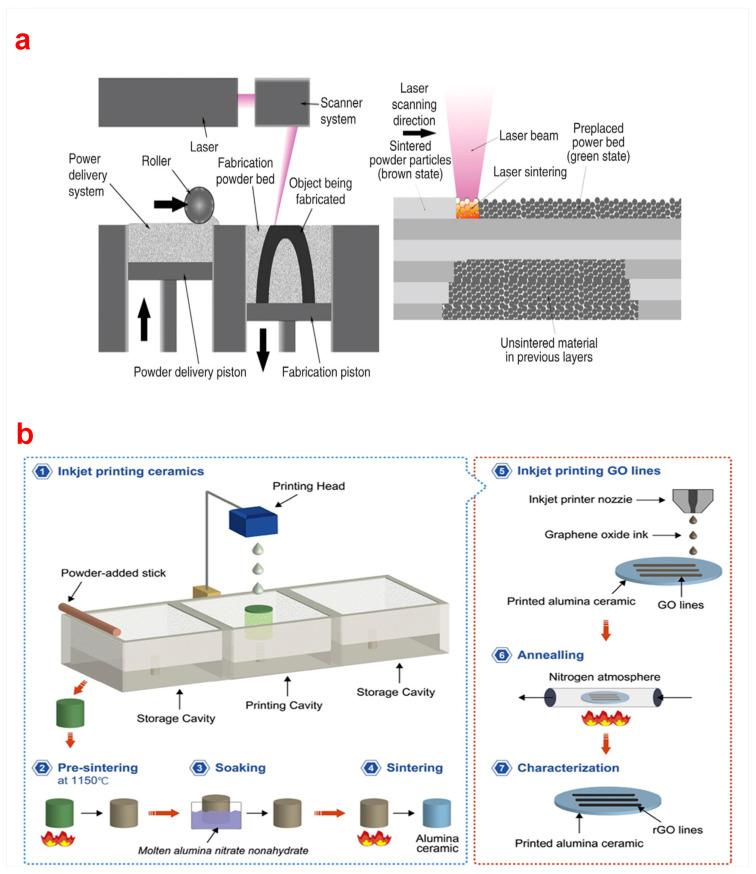
(**a**) Selective Laser Sintering [90], (**b**) printing process of bioceramics via thermal bubble inkjet technology [97].

Bioceramics and scaffolds printed via SLS have the advantages of being self-supporting, having high precision, and being in customized shapes and sizes such as lattice, honeycomb, corrugated, and sandwich structures [9]. The working principle of SLS printing is shown in Figure 5a [90]. Bioactive borate glass (BBG) has bioactivity similar to 45S5 bioactive glass, and its surface can rapidly form apatite in simulated body fluid (SBF) within 6 h [91]. The bioactive borate glass/polycaprolactone composite scaffolds with biodegradability prepared by SLS exhibited excellent in vivo biological properties due to the printed internal porous structure [92]. Shuai et al. [93] prepared the porous CaP scaffolds with various weight ratios of TCP/HA powders through SLS. The compressive strength of the scaffolds with a ratio of 30/70 was 18.35 MPa. Additionally, high-strength Al_2_O_3_ parts were fabricated via slurry-based SLS of polymer-coated ceramic powders [94]. Bulina et al. [32] fabricated HA bioceramic scaffolds via SLS. The spot size, laser power, and scanning speed of HA powder under laser radiation were 0.2 mm, 4 W, and 640 mm/s, respectively, in the process of sintering. A dense layer of HA can be formed with different compositions of apatite powder. The high brittleness of bioceramics was also overcome by SLS, which has a great improvement over traditional technologies such as fiber bonding, melt casting, and freeze drying [95].

In addition, post-processing of the printed parts is usually required to improve their surface quality and mechanical properties after SLS. This may involve additional steps such as sanding, polishing, or applying coatings, which can be time consuming and add to the overall production cost.

### 2.6. Selective Laser Melting (SLM)

SLM is a 3D technology developed to prepare scaffolds with complex structures and large sizes and offers a promising improvement for bone implant applications [7]. Alumina and silica are commonly used bioceramic materials in SLM processes. Liu et al. [96] prepared highly dense Al_2_O_3_/GdAlO_3_/ZrO_2_ ternary eutectic ceramic parts with complex shapes through SLM. The thermal bubble inkjet method and high-temperature sintering were used to print Al_2_O_3_ and graphene circuits-on-ceramics [97]. The bioceramic printing process via thermal bubble inkjet technology is shown in Figure 5b, which can economically and efficiently print Al_2_O_3_ ceramics.

In considering these advancements in scaffold fabrication, the mechanical property of the printed bioceramics or scaffolds is one of the primary concerns in order to provide enough support at an implanted site. Different printing techniques and post-processing (such as debinding and sintering) have a significant impact on the properties of the printed bioceramics and scaffolds. The bioceramic scaffolds with different sizes were manufactured through DLP; the Al_2_O_3_-ZrO_2_ composite scaffold exhibits a high flexural resistance and elastic modulus [78]. The structural and mechanical stability of porous bioceramics can be achieved by optimizing the pore size and geometry. The compressive strength (0.6–16.8 MPa) of β-TCP bioceramic scaffolds were tuned to match the needs for various locations of cancellous bone [82]. In addition, it was found that the compressive strength of HA scaffolds decreased with the increase of pore size; the scaffold with ~600 μm pores had a promising application prospect. The HA and ZrO_2_ scaffolds printed by DLP showed different compressive strength, Vickers hardness, and fracture toughness. Sintering temperature is also one of the main factors that affect the mechanical properties of bioceramics. The compressive strength (149.54 ± 20.38 MPa) of BCP bioceramics sintered at 1250 °C was significantly lower than that at 1300 °C (370.03 ± 41.24 MPa) [84].

Bioceramic scaffolds fabricated through 3D printing exhibit the following advantages: (1) 3D printing provides precise control over both internal and external architectures, including pore geometry, porosity, and interconnectivity, enables high structural complexity, design flexibility, and patient-specific requirements [98]. (2) Optimized 3D-printed scaffolds can provide growth-guiding structures to support cell migration and proliferation, thereby facilitating regenerative bone tissue formation [99]. (3) The 3DP process allows for rapid fabrication while minimizing experimental errors [100]. Based on ISO/ASTM 52900:2021 and widely used 3D printing methods for bioceramics in the literature, the advantages and disadvantages for 3D printing of bioceramics are shown in Table 1.

As shown in Table 1, DIW and SLA have been widely used in the printing of bioceramics and bioceramic matrix composite scaffolds. Although DIW needs highly controlled printing slurries and the printed parts have low accuracy, it excels in the printing of bioceramics and bioceramic matrix composite scaffolds with biomimetic structures [102]. Continuous fiber-reinforced bioceramic matrix composite scaffolds can be produced to improve the mechanical property by DIW. DLP and SLA can fabricate high-precision bioceramic scaffolds [106,116], but the addition of photosensitive resins may reduce their bioactivity, which will influence the biocompatibility of the bioceramic scaffolds. Although SLS and SLM can produce diverse bioceramic scaffolds [119] and bioactive glass components [108] without introducing additives, their limited material compatibility poses a challenge. Additionally, SLM usually requires additional support materials when it creates bioceramic scaffolds. The 3D printing method exhibits distinct advantages and should be appropriately selected to fabricate various implants of bioceramics and bioceramic matrix composite scaffolds. These different 3D printing methods would provide great potential for bone repair, bone tissue regeneration, and biomedical applications, particularly for some complex-shaped pathological bone defect conditions [120]. Additionally, the primary factors, including the 3D printing process, material powder composition and morphology, and post-processing techniques, can possibly influence the microstructure formation of scaffolds. Nevertheless, 3D-printed bioceramic scaffolds with proper in vivo degradability and excellent mechanical properties remain a challenge.

## 3. Three-Dimensional Printing of Bioceramic Matrix Composites

Due to the inadequate mechanical properties or biological properties of printed bioceramics or scaffolds by 3D printing, reinforcements (fibers, particles, whiskers, ionic doping) are introduced to improve their mechanical properties and biological activity. The mechanical properties of the composite stent should be matched to the bone at the implant site to avoid the stress shielding effect or the lack of performance of the implant. In addition, the biodegradation rates of composite scaffolds can be matched with the appropriate rate for the gradual transfer of loads from implants to newly developed bone tissues and can eliminate the need for additional surgeries to remove the scaffold, reducing the risk of complications and improving patient outcomes.

### 3.1. Fiber Reinforcements

As a reinforcing material, the addition of fibers in bioceramics can enhance their strength and toughness, thus increasing their load-bearing capacity and resistance to fracture. This can be attributed to the improved microstructural integrity and the efficient stress transfer by the added fibers. Carbon fiber (CF) has great potential to reinforce bioceramics due to its biocompatibility and high strength and modulus. CF-reinforced bioceramics have been widely used for the fabrication of bioceramic components with excellent mechanical properties [121]. Continuous fiber-reinforced bioceramics can be printed on the condition that the precursors have excellent printability [120]. Continuous carbon fiber-reinforced polymer composites were fabricated by vat photopolymerization. Zhao et al. [122] used a self-designed 3D printer to fabricate continuous CF-reinforced HA composite (CF/HA) scaffolds with simultaneous improvement in strength and toughness. Although carbon fiber reinforcement improves the strength and toughness of bioceramics, its bioinert nature remains unavoidable and can significantly reduce the biological activity of scaffolds [123]. Thus, developing novel degradable fibers to enhance bioceramics poses a critical challenge in current research.

### 3.2. Particle Reinforcements

Glass is one of the particle reinforcement materials that is used to reinforce bioceramics, and silicate bioactive glass has been widely used for biomedical applications. Bioceramic glass has unique bone-bonding properties compared to other bioceramics [124]. Bioactive glass (BG) is primarily composed of Na_2_O, CaO, SiO_2_, and P_2_O_5_, exhibiting significant potential for healing and regenerating bone defects [125]. Its osteoconductive and osteogenic properties enhanced the proliferation and differentiation of progenitor cells [126]. Additionally, BG served as a viable alternative to other scaffold biomaterials due to its ability to promote angiogenesis under vascular endothelial growth factor (VEGF) stimulation [127]. BG also helped to form carbonated hydroxyapatite and bioactive surface layers, enabling interfacial bonding with surrounding tissues [90]. Bioactive ceramics and glasses can be combined with polymers to form composites with improved mechanical properties for bone regeneration. Three-dimensional biopolymer–glass composites with introduced cells have been developed to guide cell growth, proliferation, and differentiation [128]. Hence, BG scaffolds act as an excellent porous template with outstanding mechanical performance in the fields of tissue engineering and regenerative medicine [129]. Bose et al. [130] utilized a binder jetting 3D printing method to produce bioactive glass (45S5 BG)-reinforced TCP scaffolds. The HA apatite layer was enhanced with the addition of BG in 8 weeks in vitro. In addition, Kolan et al. [131] fabricated borate-based BG scaffolds with various porosities and architectures (cubic, spherical, crossed, gyroid, and diamond) using indirect SLS. In particular, after being implanted in a rat calvarial defect, scaffolds with a diamond structure and 50% porosity exhibited faster bone formation. Porous 45S5 bioactive glass–ceramic scaffolds prepared through SLS exhibited higher density, higher crystallinity, and fracture toughness [132]. By rapidly heating and cooling, the desirable crystallization phase Na_2_Ca_2_Si_3_O_9_ was formed. The β-TCP ceramic scaffolds had excellent biocompatibility to be applied for bone regeneration. Porous scaffolds of 58S BG/β-tricalcium phosphate (β-TCP/BG) manufactured through DLP had an increased compressive strength with the increase in solid loading [133]. The β-TCP/BG composite scaffolds can promote the attachment and osteogenic differentiation of bone marrow stem cells.

**Figure 6:**
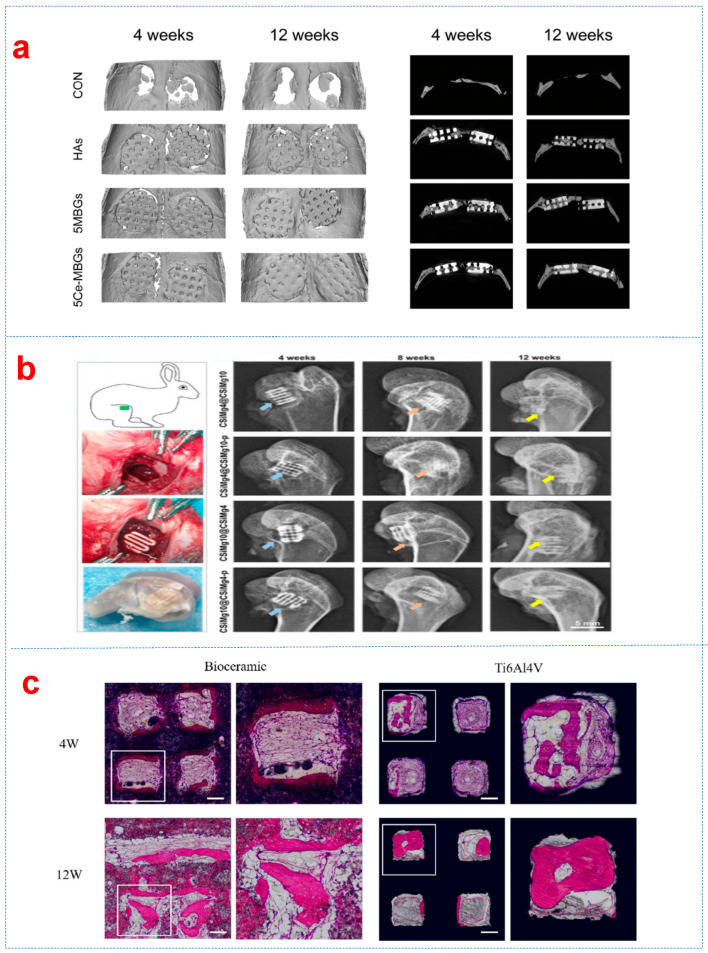
(**a**) Reconstruction micro-CT images showing the scaffolds of the HAs, 5MBGs, and 5Ce-MBGs groups implanted in rat calvarial defects at 4 and 12 weeks post-implantation [135]. (**b**) The behavior of magnesium-doped SiC scaffolds with different magnesium content after implantation [136]. (**c**) Histological sections of VG staining at 4 and 12 weeks following the implantation of various stents into a rabbit skull. The stents are shown in black, new bone in red, and fibrous tissue in blue. The length of the ruler is 300 μm [147].

In addition to BG, MgO has also been considered as a particle reinforcement material for bioceramic scaffolds due to its exceptional biocompatibility and antibacterial properties. The biodegradation of biphasic calcium phosphate (BCP) in simulated body fluid (SBF) was effectively improved when 1 wt% MgO was introduced [134]. The macroporous dome-like meshes with varying mass ratios of CSi/CSi-Mg6 were prepared through DLP using wollastonite bioceramic without and with 6% magnesium addition (CSi, CSi-Mg6) [65]. The biodegradation process and the new bone growth of the CSi/CSi-Mg6 scaffolds and the titanium mesh are shown in Figure 3c. HA bone grafts degraded slowly when extending the implantation time, providing a certain spatial maintenance for further bone growth, while the degradability of bioceramic scaffolds containing 16% CSi can be significantly improved, and the scaffolds can accelerate the growth of new bone. The biodegradability of bioceramic scaffolds can be greatly improved with the addition of baghdadite [66]. Zhao et al. [135] improved 3D-printed hydroxyapatite (HA) scaffolds by integrating cerium-containing mesoporous bioactive glass nanoparticles (Ce-MBGNs). The results showed that incorporating Ce-MBGNs significantly enhanced the HA scaffolds’ mechanical strength, mineralization capacity, antioxidant activity, and osteogenic potential. Micro-CT results showed the impact of scaffold implantation on bone growth at the defect site at 4 and 12 weeks post-implantation. Compared with other groups, 5CeMBG displayed the most significant bone formation, particularly within interconnected macropores (Figure 6b) [136]. This strategy demonstrates promising potential for developing personalized and more effective bone defect repair solutions, particularly in challenging clinical cases involving inflammatory conditions.

### 3.3. Whisker Reinforcements

Whisker reinforcements such as HA and SiC whiskers are easily mixed with bioceramics and are widely used to enhance the properties of HA, Al_2_O_3_, ZrO_2_, and Si_3_N_4_ [137,138,139]. As a strengthening agent, the whiskers prevent catastrophic failure by obstructing crack deflection and dispersing the stress concentration. Using the 3D printing method, the mixture of HA powder and whiskers (5%, 10%, and 15%) was used to prepare a porous bone embryo scaffold that had the highest compressive strength with 10% HA whiskers [140]. Feng et al. [141] used HA whiskers to reinforce a calcium silicate ceramic and fabricated the porous calcium silicate scaffolds through SLS, which can overcome insufficient mechanical strength and resistance [142]. Similarly, a SiC whisker (SiCw) with exceptional comprehensive properties, including high strength, chemical stability, and biological properties, was utilized to reinforce nHA composites [143,144]. Xing et al. [145] prepared SiC-toughened Al_2_O_3_ (SiCw/Al_2_O_3_) ceramic parts with complex shapes by using SLA. The composite ceramics demonstrated a combination of intergranular and transgranular fracture modes. The fracture toughness was enhanced owing to SiC whisker pull out and crack deflection.

### 3.4. Ionic Doping Reinforcements

Ionic doping can modulate the crystalline structure of bioceramics and is regarded as one of the effective strategies to enhance their mechanical and functional properties. In terms of biological performance, ionic doping may promote cell proliferation, differentiation, and antibacterial efficacy [146]. In the rabbit femoral bone defect repair model, three-dimensional CT reconstruction and histological observations demonstrated that the CSiMg4@CSiMg10-p scaffold exhibited significantly higher osteogenic capability compared to other scaffolds after 12 weeks of implantation (Figure 6a). The biodegradable characteristic of bioceramic scaffolds is crucial for their successful application in tissue engineering, which promotes natural healing processes and eliminates the need for scaffold removal surgeries. After 4 and 12 weeks of implantation in rabbit skulls, Mg-doped calcium silicate (CSM) bioceramic scaffolds and titanium alloy scaffolds demonstrated differences in bone tissue ingrowth. At the 4th week, more bone tissue cells adhered to the inner wall and penetrated the pores within the scaffold compared with the titanium alloy scaffold [147]. Figure 6c displays histological sections stained with VG at 4 and 12 weeks. The CSM bioceramic scaffold exhibited gradual degradation with numerous bone tissue cells aligning along the inner wall of the scaffold. At the 12th week, new bone tissue had extended along the inner wall and into the interior. The scaffolds exhibited excellent osseointegration ability by fusing with bone tissue to create a robust interface between the scaffold and bone. However, the compressive strength of the CSM bioceramic scaffold decreased rapidly with in vivo degradation, which was not conducive to repairing load-bearing bone defects.

Ion-doped bioceramic scaffolds exhibit significant potential in immune modulation by releasing specific bioactive ions to enhance bone regeneration [148]. However, the synergistic effects between ions and their underlying interaction mechanisms require further investigation, while the precision and stability of ion doping ratios and distribution need improvement.

A hierarchical structure serves as a framework defining the structure–function relationships in natural bone, including cortical bone and cancellous bone. Various mechanical, biological, and chemical functions are carried out by macro-, micron-, and nanoscale structures. A stabilizing and supportive role is provided by cortical bone with notable mechanical strength, while cancellous bone with 50% to 90% porosity provides a proper environment for bone metabolism and hematopoietic function [149]. Inspired by the structure and composition of natural bone, bone substitute and repair materials should have a hierarchical and anisotropic structure and composition similar to natural bone to achieve desirable biological and mechanical performances. Such scaffolds for bone tissue engineering should contain multilevel pores from the micro- to the macro-scale, which facilitate bone regeneration and vascular ingrowth and can be applied for both non-load-bearing and load-bearing bone defects. Implanted bioceramics can interact with the surrounding tissues and stimulate specific cellular responses. This interaction can create a chemical bond between the implant and the tissue, promoting integration and long-term stability [150]. An interconnected pore structure in a scaffold can allow oxygen, nutrients, and waste to be delivered through the scaffold. A scaffold with high porosity has a large surface area, which will improve cell adhesion, cell migration, and tissue–scaffold interaction. Additionally, an ideal scaffold should have similar mechanical properties (including strength, stiffness, and toughness) to natural bone. It can provide a certain mechanical support and deliver it to the cell under physiological conditions, which can stimulate cell differentiation from osteogenic lineages into undesirable forms. It has been reported that some scaffolds achieved comparable mechanical properties to those of cancellous bone, which will provide enough mechanical support in repairing non-load-bearing bone defects. Nevertheless, few scaffolds possess similar strength, stiffness, and toughness to that of cortical bone. Their mechanical weaknesses, including non-ideal strength and toughness, restrict them from repairing load-bearing bones. Continuous monofilament fiber-reinforced bioceramics can obtain satisfying microstructure and mechanical properties for both the cancellous and cortical bone. However, 3D printer and printing technology can hardly prepare a continuous fiber-reinforced bioceramic scaffold with fine structure and mechanical properties because monofilament is not easily centered on, adjusted with, fed into, and bonded with the matrix in a nozzle during the process of printing. The adhesion performance between the continuous fiber and bioceramic matrix is weak, which seriously reduces the mechanical properties of the composite scaffold. This limits the clinical application of monofilament fiber-reinforced bioceramics mimicking natural bone in both microstructure and mechanical properties. Moreover, besides the common reinforcing materials, such as carbon fiber and glass fibers, few kinds of degradable fiber can be used to reinforce degradable and absorbable bioceramics for clinical application.

## 4. Summary and Outlook

Three-dimensional printing technology has been used to print hearts, kidneys, and artificial bone skulls, which can be used to replace bone skulls with damage and defect according to the specific conditions of patients. Three-dimensional-printed bioceramic scaffolds are especially highly suitable for bone repair applications owing to excellent biocompatibility, excellent osseointegration, excellent osteoconduction, excellent bioactivity, excellent personalized customization, and high accuracy. Direct ink writing technology with its unique advantages, such as good biocompatibility, material-saving properties, ease of operation, and fast-forming properties, has become one of the most promising 3D printing technologies in the biomedical field. Unfortunately, it is difficult to print bioceramics with high solid loading due to the lack of an excellent slurry with a low viscosity and high solid content for fabricating bioceramic green bodies in the direct writing molding process. The influence of the morphology and structure of ceramic powder on slurry characteristics, forming, and printing needs to be systematically investigated to obtain bioceramic scaffolds with high solid loading.

Furthermore, bone substitute and repair materials should have a hierarchical and anisotropic structure and composition similar to natural bone to achieve desirable biological and mechanical performance. For bone tissue engineering, scaffolds should contain multilevel pores from the micro- to the macro-scale and have satisfactory mechanical properties (including strength, stiffness, and toughness) for non-load-bearing and load-bearing bone repair applications. So far, some scaffolds achieved the mechanical properties comparable to that of cancellous bone, which were applied in repairing non-load-bearing bone defects. Nevertheless, few scaffolds possess similar strength, stiffness, and toughness to that of cortical bone. The implanted bioceramic components can be significantly enhanced by introducing different reinforcements. Continuous monofilament fiber-reinforced bioceramics can obtain satisfying microstructure and mechanical properties for both the cancellous and cortical bone. However, 3D printer and printing technology can hardly prepare a continuous fiber-reinforced bioceramic scaffold with fine structure and ideal mechanical properties because monofilament is not easily centered on, adjusted to, fed into, and bonded with the matrix in a nozzle during the printing process. The weak adhesion performance between the continuous fiber and bioceramic matrix seriously reduces the mechanical properties of the composite scaffold. Moreover, besides the common reinforcing materials such as carbon fiber and glass fiber, there are few kinds of degradable fibers for reinforcing degradable and absorbable bioceramics in clinical applications. Monofilament fiber-reinforced bioceramics mimicking natural bone in both microstructure and mechanical properties can be a solution in load-bearing applications, which urgently need research and development of the printer and novel technology for monofilament fiber-reinforced bioceramics. The monofilament fiber-reinforced bioceramic scaffolds with proper in vivo degradability will promote natural healing processes and eliminate the need for scaffold removal surgeries, which remain a challenge.

Although existing research has significantly improved the performance of traditional scaffolds, 3D-printed bioceramic scaffolds remain far from clinical application due to persistent challenges. First, current 3D printing technologies struggle to fabricate bioceramic scaffolds with both high strength and toughness. While high-temperature sintering is commonly used to produce high-strength ceramic scaffolds, their purely ceramic composition leads to insufficient toughness and fracture susceptibility in practical use. These limitations prevent 3D-printed bioceramic scaffolds from adapting to the unique mechanical demands of load-bearing skeletal environments, thereby restricting their applicability compared to metal implants. Second, the degradation rate of existing bioceramics often fails to align with new bone formation rates, hindering optimal bone-scaffold integration. Finally, current 3D-printed bioceramic scaffolds cannot accurately replicate the highly complex and ordered microstructure of natural bone tissue. These issues represent critical challenges in 3D printing of bioceramics. To address these challenges, future research should focus on refining existing 3D printing technologies to enhance precision for fabricating finer and more intricate structures, improving control over scaffold microarchitecture to mimic the composition and structure of natural high-strength/toughness materials (e.g., bone, nacre), and developing multi-material composite bioceramic scaffolds with superior mechanical properties and tunable degradation rates that match bone regeneration kinetics. Although 3D-printed bioceramic scaffolds are still largely confined to laboratory research, they present both significant opportunities and formidable challenges for clinical translation. This will be a significant opportunity and formidable challenge for clinical application.

## Figures and Tables

**Table 1:** Advantages and disadvantages for 3D printing of bioceramics.

3D Printing Methods	Advantages	Disadvantages	Materials
Direct Ink Writing (DIW)	Suitable for high solids slurries; Flexible structural design; Low cost	Slow printing speed; The rheological properties of the slurry are required	Bioactive glass [100] HAp [101] CSi-Mg [102] β-TCP [27]
Digital Light Processing (DLP)	Fast printing speed; High precision;	Add photosensitive resin; Limited materials; Post-processing is required	HA [103] Al_2_O_3_ [104] ZrO_2_ [68] β-TCP [105] Bioactive glass [106]
Selective Laser Melting (SLM)	High molding density; Excellent mechanical properties	Expensive equipment; Only suitable for metals or ceramics with high melting points	Bioactive glass [107] CaO_3_Si [108]
Fused Deposition Manufacturing (FDM)	Equipment of low cost; Simple operation; Multi-material printing	Low accuracy; Weak inter-layer adhesion	PCL/HA [109] PLA/HA [110] PCL/β-TCP [111] PLA/β-TCP [112]
Selective Laser Sintering (SLS)	No support structure; Suitable for porous and complex geometries; High utilization	The high surface roughness; Post-treatment; High-temperature sintering lead to grain coarsening	Bioactive glass [113] β-TCP [114] HA/β-TCP [115]
Stereolithography (SLA)	High resolution; Suitable for fine structures	High cost; Material selection is restricted; Post-processing is required	Bioactive glass [116] Al_2_O_3_ [117] ZrO_2_ [118] HA [119] β-TCP [82]
